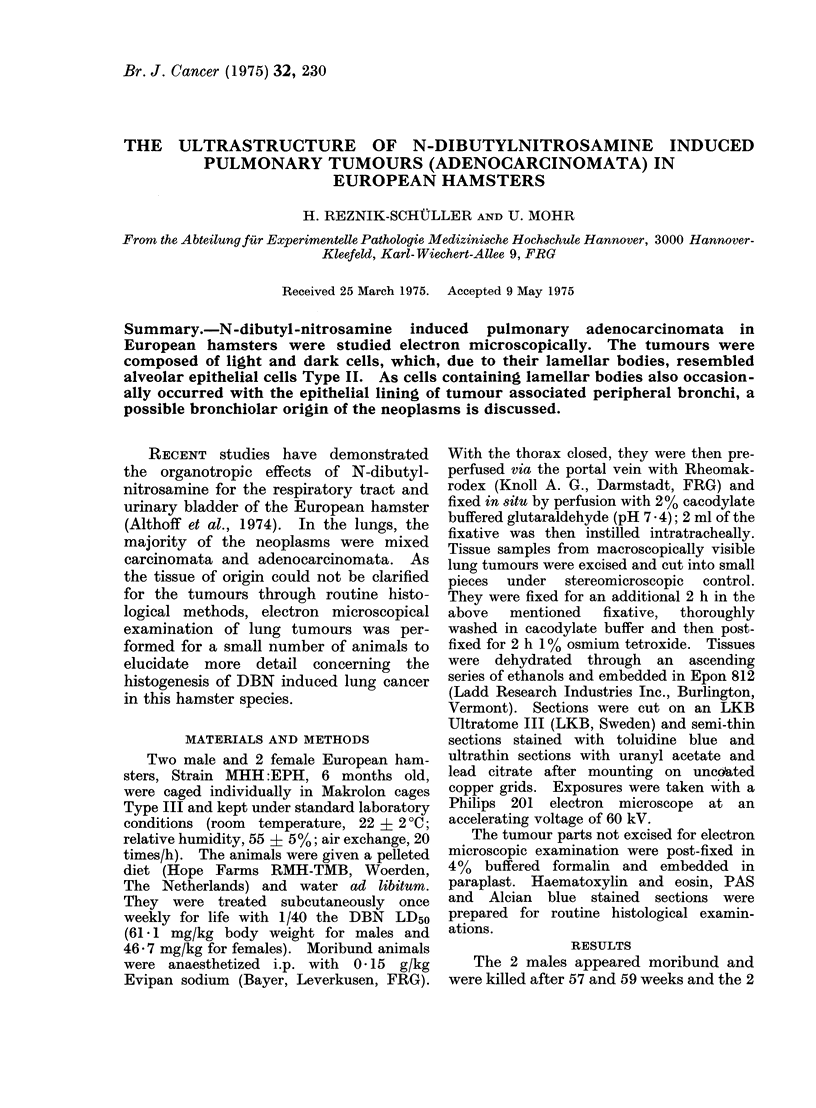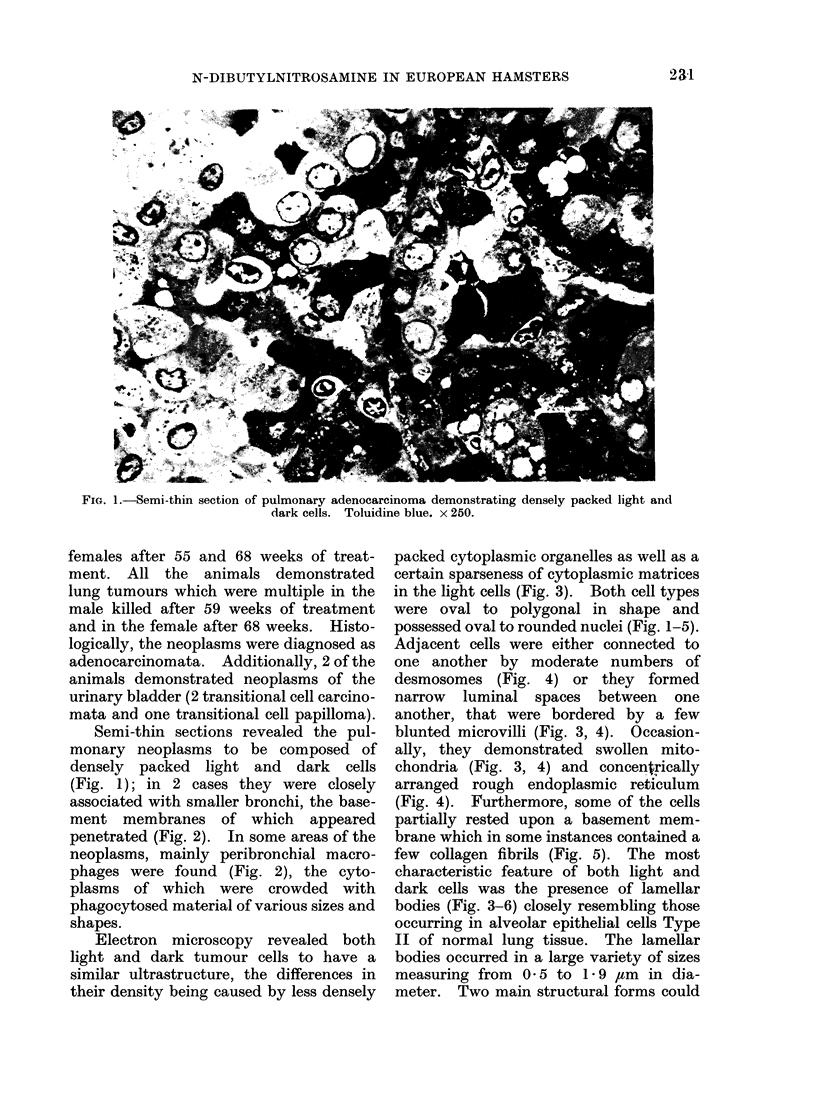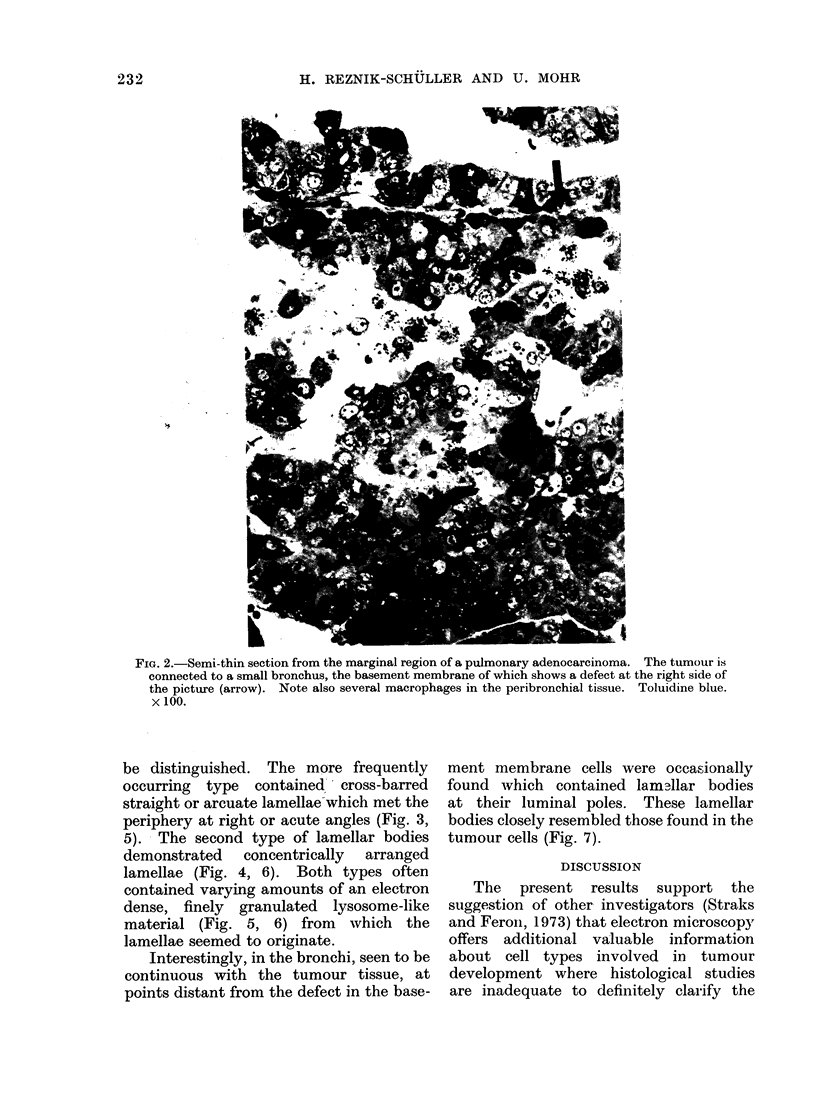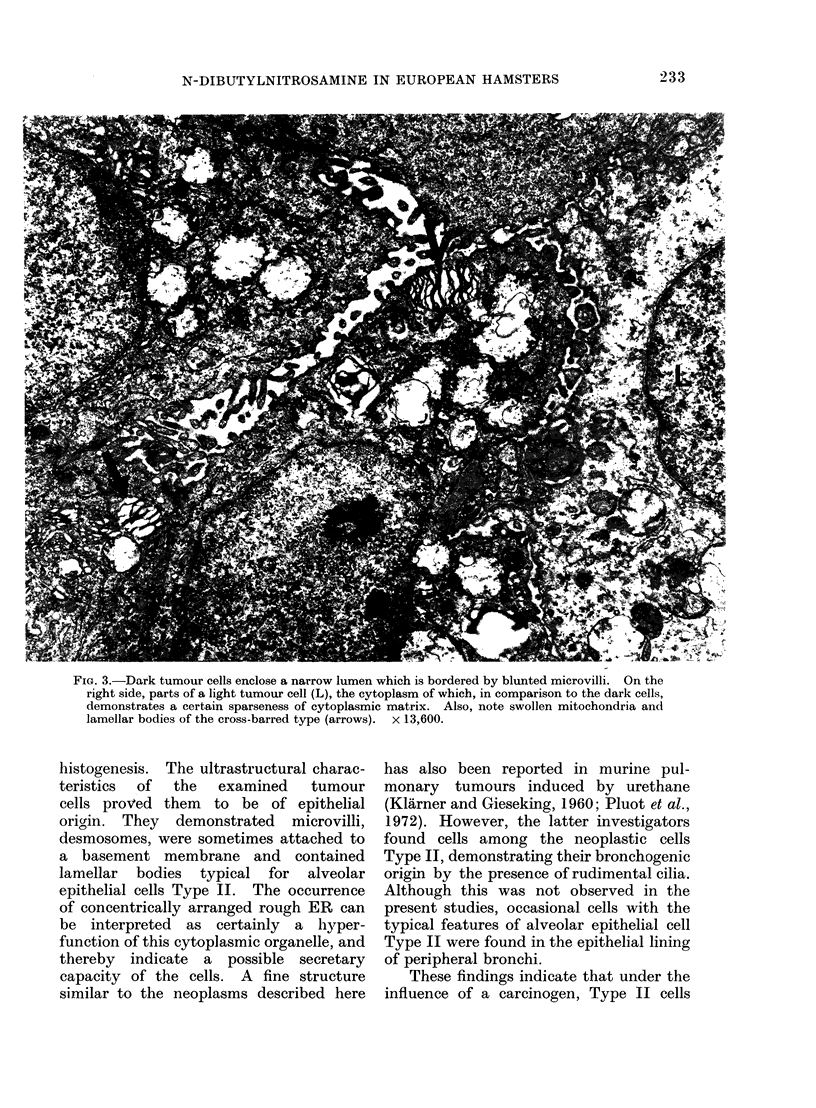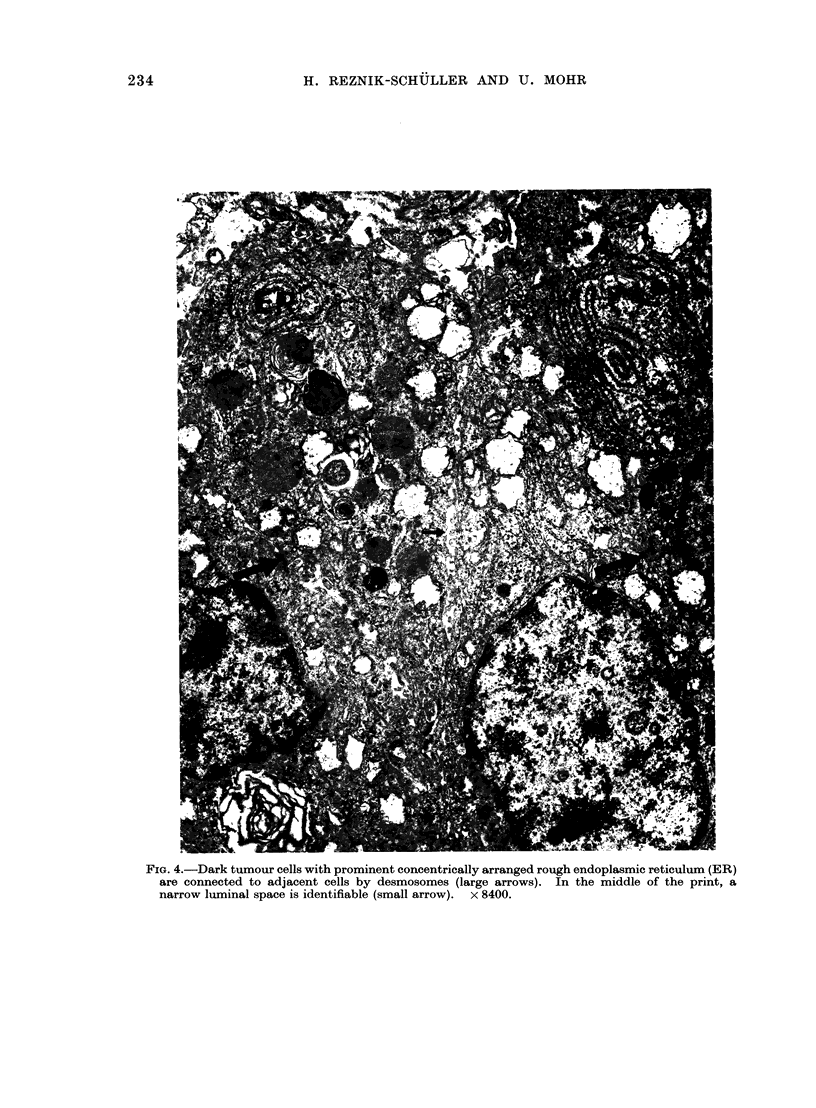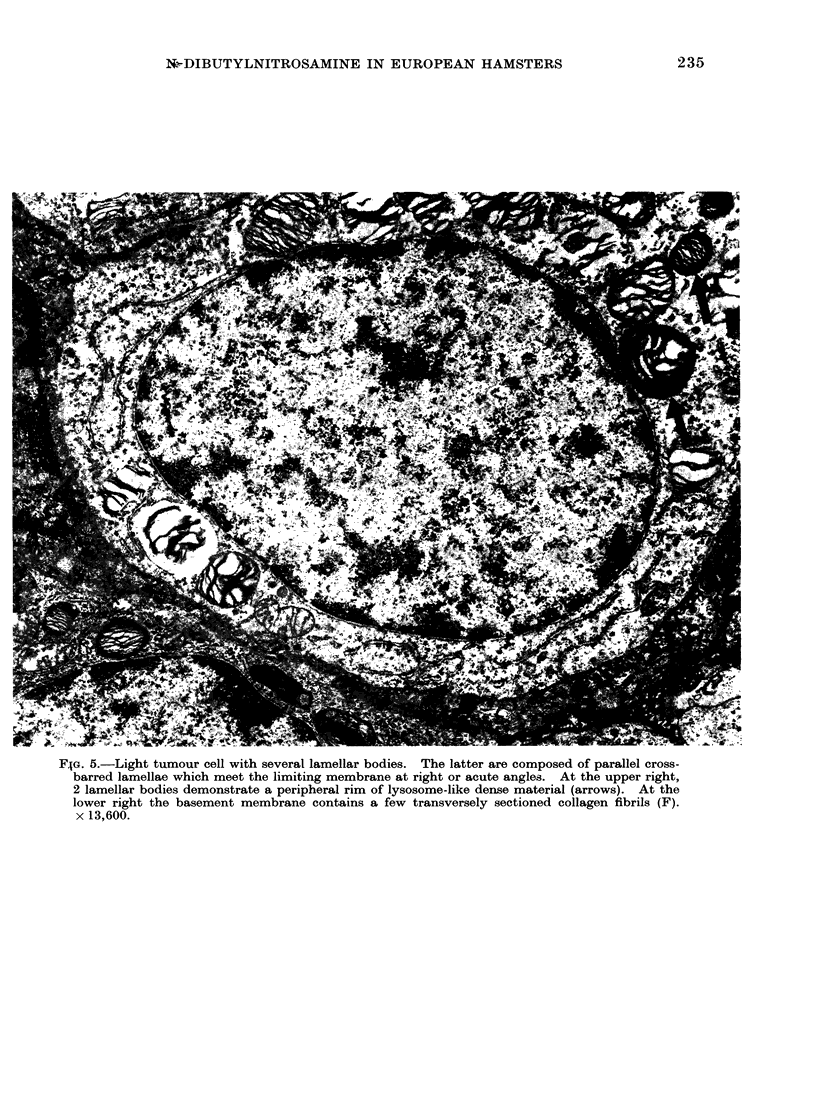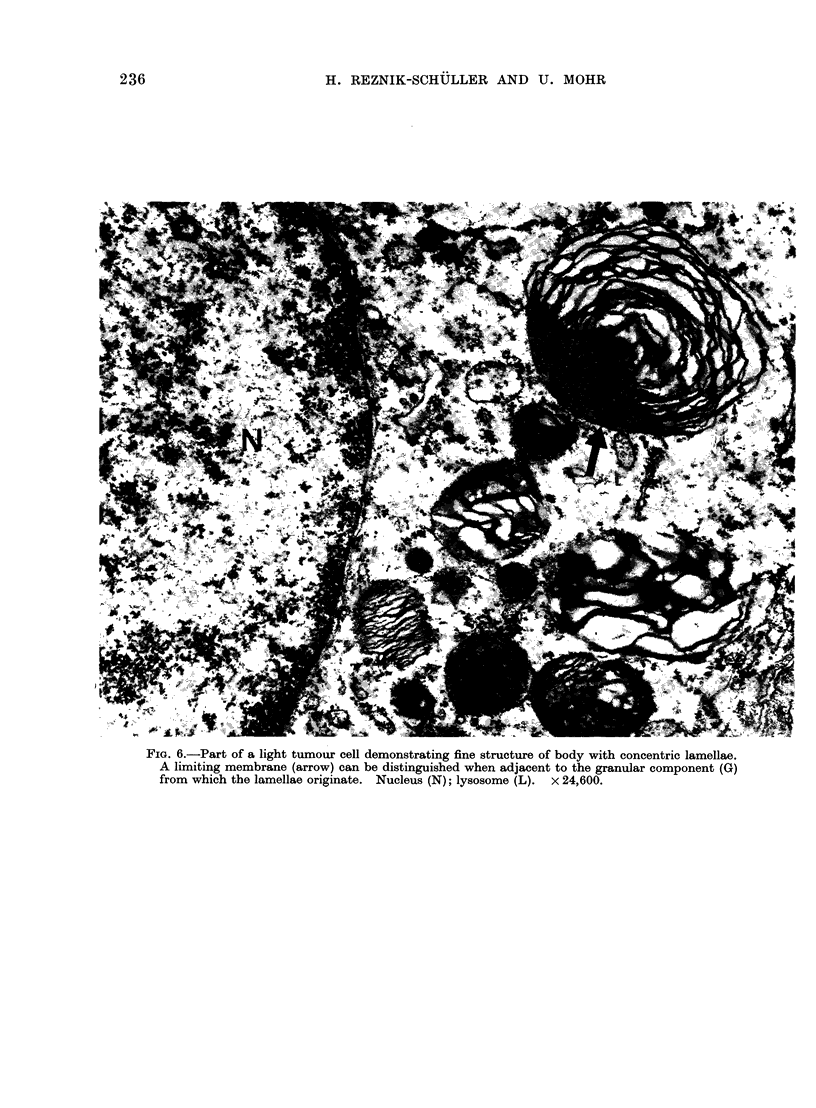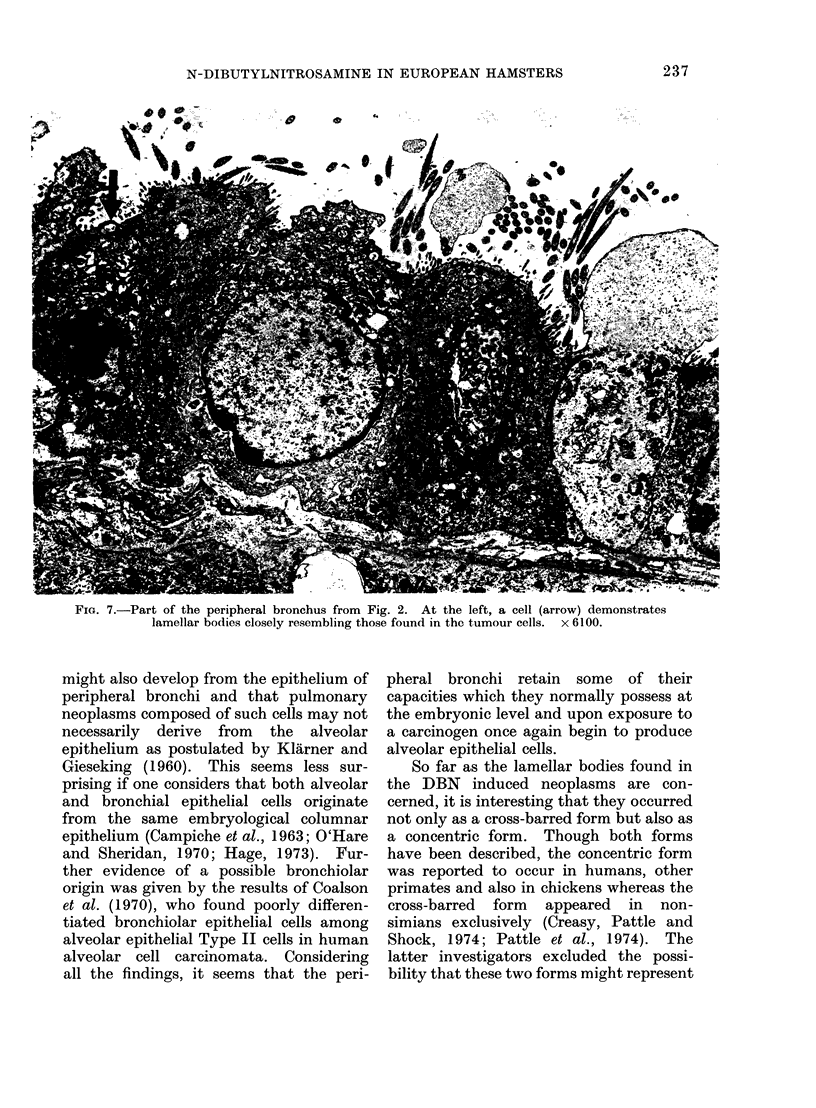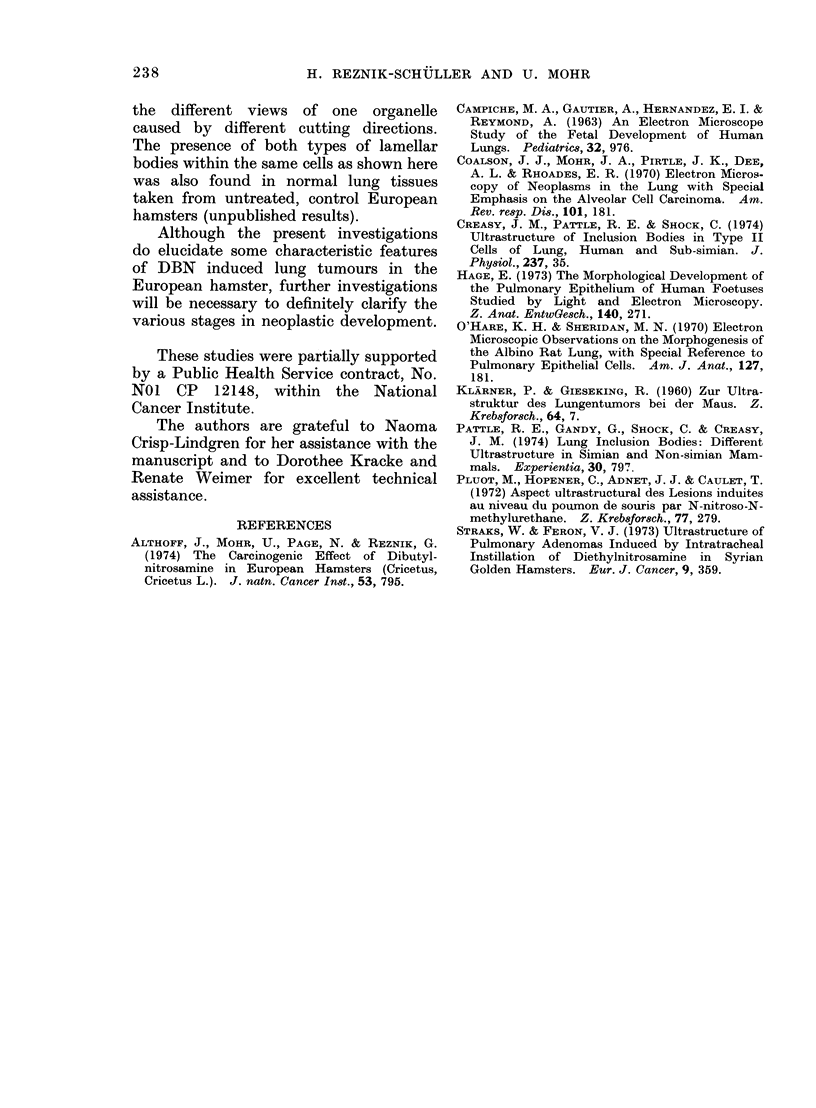# The ultrastructure of N-dibutylnitrosamine induced pulmonary tumours (adenocarcinomata) in European hamsters.

**DOI:** 10.1038/bjc.1975.153

**Published:** 1975-08

**Authors:** H. Reznik-Schüller, U. Mohr

## Abstract

**Images:**


*Br. J. Cancer* (1975) **32**, 230

### THE ULTRASTRUCTURE OF N-DIBUTYLNITROSAMINE INDUCED PULMONARY TUMOURS (ADENOCARCINOMATA) IN EUROPEAN HAMSTERS

H. REZNIK-SCHÜLLER AND U. MOHR

*From the Abteilung für Experimentelle Pathologie Medizinische Hochschule Hannover*, 3000 *Hannover-Kleefeld, Karl-Wiechert-Allee* 9, *FRG*

Received 25 March 1975. Accepted 9 May 1975

**Summary.—N-dibutyl-nitrosamine induced pulmonary adenocarcinomata in European hamsters were studied electron microscopically. The tumours were composed of light and dark cells, which, due to their lamellar bodies, resembled alveolar epithelial cells Type II. As cells containing lamellar bodies also occasionally occurred with the epithelial lining of tumour associated peripheral bronchi, a possible bronchiolar origin of the neoplasms is discussed.**

RECENT studies have demonstrated the organotropic effects of N-dibutyl-nitrosamine for the respiratory tract and urinary bladder of the European hamster (Althoff *et al.*, 1974). In the lungs, the majority of the neoplasms were mixed carcinomata and adenocarcinomata. As the tissue of origin could not be clarified for the tumours through routine histological methods, electron microscopical examination of lung tumours was performed for a small number of animals to elucidate more detail concerning the histogenesis of DBN induced lung cancer in this hamster species.

### MATERIALS AND METHODS

Two male and 2 female European hamsters, Strain MHH:EPH, 6 months old, were caged individually in Makrolon cages Type III and kept under standard laboratory conditions (room temperature, $22 \pm 2\,^{\circ}C$; relative humidity, $55 \pm 5\%$; air exchange, 20 times/h). The animals were given a pelleted diet (Hope Farms RMH-TMB, Woerden, The Netherlands) and water *ad libitum*. They were treated subcutaneously once weekly for life with 1/40 the DBN $LD_{50}$ (61·1 mg/kg body weight for males and 46·7 mg/kg for females). Moribund animals were anaesthetized i.p. with 0·15 g/kg Evipan sodium (Bayer, Leverkusen, FRG). With the thorax closed, they were then preperfused *via* the portal vein with Rheomakrodex (Knoll A. G., Darmstadt, FRG) and fixed *in situ* by perfusion with 2% cacodylate buffered glutaraldehyde (pH 7·4); 2 ml of the fixative was then instilled intratracheally. Tissue samples from macroscopically visible lung tumours were excised and cut into small pieces under stereomicroscopic control. They were fixed for an additional 2 h in the above mentioned fixative, thoroughly washed in cacodylate buffer and then post-fixed for 2 h 1% osmium tetroxide. Tissues were dehydrated through an ascending series of ethanols and embedded in Epon 812 (Ladd Research Industries Inc., Burlington, Vermont). Sections were cut on an LKB Ultratome III (LKB, Sweden) and semi-thin sections stained with toluidine blue and ultrathin sections with uranyl acetate and lead citrate after mounting on uncoated copper grids. Exposures were taken with a Philips 201 electron microscope at an accelerating voltage of 60 kV.

The tumour parts not excised for electron microscopic examination were post-fixed in 4% buffered formalin and embedded in paraplast. Haematoxylin and eosin, PAS and Alcian blue stained sections were prepared for routine histological examinations.

### RESULTS

The 2 males appeared moribund and were killed after 57 and 59 weeks and the 2

FIG. 1.—Semi-thin section of pulmonary adenocarcinoma demonstrating densely packed light and dark cells. Toluidine blue. ×250.

females after 55 and 68 weeks of treatment. All the animals demonstrated lung tumours which were multiple in the male killed after 59 weeks of treatment and in the female after 68 weeks. Histologically, the neoplasms were diagnosed as adenocarcinomata. Additionally, 2 of the animals demonstrated neoplasms of the urinary bladder (2 transitional cell carcinomata and one transitional cell papilloma).

Semi-thin sections revealed the pulmonary neoplasms to be composed of densely packed light and dark cells (Fig. 1); in 2 cases they were closely associated with smaller bronchi, the basement membranes of which appeared penetrated (Fig. 2). In some areas of the neoplasms, mainly peribronchial macrophages were found (Fig. 2), the cytoplasms of which were crowded with phagocytosed material of various sizes and shapes.

Electron microscopy revealed both light and dark tumour cells to have a similar ultrastructure, the differences in their density being caused by less densely packed cytoplasmic organelles as well as a certain sparseness of cytoplasmic matrices in the light cells (Fig. 3). Both cell types were oval to polygonal in shape and possessed oval to rounded nuclei (Fig. 1–5). Adjacent cells were either connected to one another by moderate numbers of desmosomes (Fig. 4) or they formed narrow luminal spaces between one another, that were bordered by a few blunted microvilli (Fig. 3, 4). Occasionally, they demonstrated swollen mitochondria (Fig. 3, 4) and concentrically arranged rough endoplasmic reticulum (Fig. 4). Furthermore, some of the cells partially rested upon a basement membrane which in some instances contained a few collagen fibrils (Fig. 5). The most characteristic feature of both light and dark cells was the presence of lamellar bodies (Fig. 3–6) closely resembling those occurring in alveolar epithelial cells Type II of normal lung tissue. The lamellar bodies occurred in a large variety of sizes measuring from 0·5 to 1·9 $\mu$m in diameter. Two main structural forms could

FIG. 2.—Semi-thin section from the marginal region of a pulmonary adenocarcinoma. The tumour is connected to a small bronchus, the basement membrane of which shows a defect at the right side of the picture (arrow). Note also several macrophages in the peribronchial tissue. Toluidine blue. ×100.

be distinguished. The more frequently occurring type contained cross-barred straight or arcuate lamellae which met the periphery at right or acute angles (Fig. 3, 5). The second type of lamellar bodies demonstrated concentrically arranged lamellae (Fig. 4, 6). Both types often contained varying amounts of an electron dense, finely granulated lysosome-like material (Fig. 5, 6) from which the lamellae seemed to originate.

Interestingly, in the bronchi, seen to be continuous with the tumour tissue, at points distant from the defect in the basement membrane cells were occasionally found which contained lamellar bodies at their luminal poles. These lamellar bodies closely resembled those found in the tumour cells (Fig. 7).

### DISCUSSION

The present results support the suggestion of other investigators (Straks and Feron, 1973) that electron microscopy offers additional valuable information about cell types involved in tumour development where histological studies are inadequate to definitely clarify the

FIG. 3.—Dark tumour cells enclose a narrow lumen which is bordered by blunted microvilli. On the right side, parts of a light tumour cell (L), the cytoplasm of which, in comparison to the dark cells, demonstrates a certain sparseness of cytoplasmic matrix. Also, note swollen mitochondria and lamellar bodies of the cross-barred type (arrows). ×13,600.

histogenesis. The ultrastructural characteristics of the examined tumour cells proved them to be of epithelial origin. They demonstrated microvilli, desmosomes, were sometimes attached to a basement membrane and contained lamellar bodies typical for alveolar epithelial cells Type II. The occurrence of concentrically arranged rough ER can be interpreted as certainly a hyperfunction of this cytoplasmic organelle, and thereby indicate a possible secretary capacity of the cells. A fine structure similar to the neoplasms described here has also been reported in murine pulmonary tumours induced by urethane (Klärner and Gieseking, 1960; Pluot *et al.*, 1972). However, the latter investigators found cells among the neoplastic cells Type II, demonstrating their bronchogenic origin by the presence of rudimental cilia. Although this was not observed in the present studies, occasional cells with the typical features of alveolar epithelial cell Type II were found in the epithelial lining of peripheral bronchi.

These findings indicate that under the influence of a carcinogen, Type II cells

FIG. 4.—Dark tumour cells with prominent concentrically arranged rough endoplasmic reticulum (ER) are connected to adjacent cells by desmosomes (large arrows). In the middle of the print, a narrow luminal space is identifiable (small arrow). ×8400.

FIG. 5.—Light tumour cell with several lamellar bodies. The latter are composed of parallel cross-barred lamellae which meet the limiting membrane at right or acute angles. At the upper right, 2 lamellar bodies demonstrate a peripheral rim of lysosome-like dense material (arrows). At the lower right the basement membrane contains a few transversely sectioned collagen fibrils (F). × 13,600.

FIG. 6.—Part of a light tumour cell demonstrating fine structure of body with concentric lamellae. A limiting membrane (arrow) can be distinguished when adjacent to the granular component (G) from which the lamellae originate. Nucleus (N); lysosome (L). ×24,600.

FIG. 7.—Part of the peripheral bronchus from Fig. 2. At the left, a cell (arrow) demonstrates lamellar bodies closely resembling those found in the tumour cells. ×6100.

might also develop from the epithelium of peripheral bronchi and that pulmonary neoplasms composed of such cells may not necessarily derive from the alveolar epithelium as postulated by Klärner and Gieseking (1960). This seems less surprising if one considers that both alveolar and bronchial epithelial cells originate from the same embryological columnar epithelium (Campiche *et al.*, 1963; O'Hare and Sheridan, 1970; Hage, 1973). Further evidence of a possible bronchiolar origin was given by the results of Coalson *et al.* (1970), who found poorly differentiated bronchiolar epithelial cells among alveolar epithelial Type II cells in human alveolar cell carcinomata. Considering all the findings, it seems that the peripheral bronchi retain some of their capacities which they normally possess at the embryonic level and upon exposure to a carcinogen once again begin to produce alveolar epithelial cells.

So far as the lamellar bodies found in the DBN induced neoplasms are concerned, it is interesting that they occurred not only as a cross-barred form but also as a concentric form. Though both forms have been described, the concentric form was reported to occur in humans, other primates and also in chickens whereas the cross-barred form appeared in non-simians exclusively (Creasy, Pattle and Shock, 1974; Pattle *et al.*, 1974). The latter investigators excluded the possibility that these two forms might represent the different views of one organelle caused by different cutting directions. The presence of both types of lamellar bodies within the same cells as shown here was also found in normal lung tissues taken from untreated, control European hamsters (unpublished results).

Although the present investigations do elucidate some characteristic features of DBN induced lung tumours in the European hamster, further investigations will be necessary to definitely clarify the various stages in neoplastic development.

These studies were partially supported by a Public Health Service contract, No. N01 CP 12148, within the National Cancer Institute.

The authors are grateful to Naoma Crisp-Lindgren for her assistance with the manuscript and to Dorothee Kracke and Renate Weimer for excellent technical assistance.

### REFERENCES

Althoff, J., Mohr, U., Page, N. & Reznik, G. (1974) The Carcinogenic Effect of Dibutylnitrosamine in European Hamsters (Cricetus, Cricetus L.). *J. natn. Cancer Inst.*, **53**, 795.

Campiche, M. A., Gautier, A., Hernandez, E. I. & Reymond, A. (1963) An Electron Microscope Study of the Fetal Development of Human Lungs. *Pediatrics*, **32**, 976.

Coalson, J. J., Mohr, J. A., Pirtle, J. K., Dee, A. L. & Rhoades, E. R. (1970) Electron Microscopy of Neoplasms in the Lung with Special Emphasis on the Alveolar Cell Carcinoma. *Am. Rev. resp. Dis.*, **101**, 181.

Creasy, J. M., Pattle, R. E. & Shock, C. (1974) Ultrastructure of Inclusion Bodies in Type II Cells of Lung, Human and Sub-simian. *J. Physiol.*, **237**, 35.

Hage, E. (1973) The Morphological Development of the Pulmonary Epithelium of Human Foetuses Studied by Light and Electron Microscopy. *Z. Anat. EntwGesch.*, **140**, 271.

O'Hare, K. H. & Sheridan, M. N. (1970) Electron Microscopic Observations on the Morphogenesis of the Albino Rat Lung, with Special Reference to Pulmonary Epithelial Cells. *Am. J. Anat.*, **127**, 181.

Klärner, P. & Gieseking, R. (1960) Zur Ultrastruktur des Lungentumors bei der Maus. *Z. Krebsforsch.*, **64**, 7.

Pattle, R. E., Gandy, G., Shock, C. & Creasy, J. M. (1974) Lung Inclusion Bodies: Different Ultrastructure in Simian and Non-simian Mammals. *Experientia*, **30**, 797.

Pluot, M., Hopener, C., Adnet, J. J. & Caulet, T. (1972) Aspect ultrastructural des Lesions induites au niveau du poumon de souris par N-nitroso-N-methylurethane. *Z. Krebsforsch.*, **77**, 279.

Straks, W. & Feron, V. J. (1973) Ultrastructure of Pulmonary Adenomas Induced by Intratracheal Instillation of Diethylnitrosamine in Syrian Golden Hamsters. *Eur. J. Cancer*, **9**, 359.